# Water-induced mantle overturn explains high Archean paleointensities

**DOI:** 10.1093/nsr/nwaf578

**Published:** 2025-12-23

**Authors:** Dong Wang, Zhongqing Wu

**Affiliations:** State Key Laboratory of Precision Geodesy, School of Earth and Space Sciences, University of Science and Technology of China, Hefei 230026, China; State Key Laboratory of Precision Geodesy, School of Earth and Space Sciences, University of Science and Technology of China, Hefei 230026, China; Mengcheng National Geophysical Observatory, University of Science and Technology of China, Bozhou 233500, China

**Keywords:** magma ocean, mantle overturn, paleomagnetic field, early earth

## Abstract

As a consequence of the evolution of the water-bearing basal magma ocean, water-induced mantle overturn can suitably explain many puzzling observations in the Archean, including the formation of continents and the Archean–Proterozoic boundary. The upwelling of the hot basal magma ocean during mantle overturn drastically affects the thermal state of the core–mantle boundary and geomagnetic field. We model the thermal evolution of the core–mantle boundary to investigate the effects of mantle overturn on the geomagnetic field. Our results demonstrate that mantle overturn substantially accelerates core cooling and increases heat flow across the core–mantle boundary. Such enhanced heat flow would have strengthened the geomagnetic field, which explains well the high paleointensity records from ∼3.5 to 2.5 Ga. The strong geodynamo and formation of Archean continents generate a concordant picture of the evolution of water-induced mantle overturn.

## Teaser

Water-induced mantle overturn accelerated core cooling and enhanced the geomagnetic field, offering a unified explanation for the high paleointensities and the emergence of continents in the Archean.

## INTRODUCTION

The Earth’s magnetic field is essential for shielding the planet from harmful solar radiation and preserving its habitability [1,2]. This field, generated by the geodynamo within the outer core, has existed for at least 3.5 billion years [3,4]. Paleointensity variations and their underlying mechanisms provide insights into Earth’s evolution, thus attracting considerable research attention [5–10]. Paleointensity data reported by Tarduno *et al.* [3,6,11–13] reveal a significant strengthening of the geomagnetic field around 3.5 Ga. From the statistical analysis of a paleointensity database (PINT) by Biggin *et al.* [14,15], the geomagnetic field in 3.5–2.5 Ga appears to be ∼30% stronger than in 2.5–1.5 Ga if uncertainties are neglected. Although the Monte Carlo Axial Dipole Average Model (MCADAM) based on the updated PINT database does not explicitly emphasize this ancient enhancement, the paleointensity in 3.5–2.5 Ga was nearly double compared to earlier and later periods [16,17] (excluding the strong Phanerozoic signal [15,18]). Ziegler and Stegman [18] highlighted this enhanced geomagnetic field, and proposed that it may have originated from a silicate dynamo within the basal magma ocean (MO) [19–21] owing to its high electrical conductivity [22,23]. However, the magnetic field generated by such silicate dynamo is expected to decay from 4.5 Ga [22]. Light element exsolution at the top of the core possibly contributes to the strong geodynamo through thermochemical convection, but the exsolution time and rate of light elements are highly uncertain [24–29]. Alternatively, the geodynamo is sensitive to the conditions at the core–mantle boundary (CMB) [30]. High paleointensity may simply reflect variations in the CMB during the long-term decay of the geodynamo, for example, due to subducted slabs reaching the CMB; however, the onset time of plate tectonics remains highly debated [13,31]. Clarifying the mechanisms underlying this enhanced geomagnetic field in the Archean is crucial for understanding Earth’s early evolution.

Recently, Wu *et al.* [32] suggested that the basal MO may have become gravitationally unstable in the late stages of crystallization because water enrichment. It is proposed that the triggered mantle overturns could have contributed to the formation of the Archean continents and subcontinental lithospheric mantle, offering potential explanations for various observations, including the rare tonalite–trondhjemite–granodiorite (TTG) in the Hadean and post-Archean periods and growing TTG emergence in the Archean [33,34]. Once water-induced mantle overturns depleted the basal MO, the mechanism of Archean continent formation changed, likely fostering the global change in the properties of continental crust at the Archean–Proterozoic boundary [32,35].

The rapid demise of the hot basal MO would have transported thermal energy from the CMB to shallower areas during mantle overturns, which would have greatly accelerated core cooling. In this study, we investigate the cooling caused by the mantle overturns, and explore their effects on the geomagnetic field to identify potential paleomagnetic records caused by the early overturning mantle.

## RESULTS AND DISCUSSION

### Water-induced mantle overturn accelerated CMB cooling

**Figure 1.**
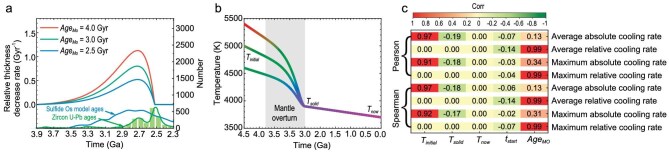
Thickness evolution of basal MO and its impact on CMB cooling. (a) Relative decrease rate in thickness of basal MO caused by mantle overturn ($\frac{{dH}}{{{H}_{{start}}dt}}$, see details in Methods). For ${T}_{{initial}}{\mathrm{\ }}$= 5000 K, ${T}_{{solid}}$ = 3900 K, ${t}_{{start}}$ = 3.9 Ga, ${T}_{now}$ = 3700 K, and $Ag{e}_{MO}$ = 2.5–4.0 Gyr. The age distribution of zircons (green curve) and sulfides in mantle-derived xenoliths and xenocrysts (blue curve) are from Griffin *et al.* [34]. (b) CMB cooling history for ${T}_{{initial}}{\mathrm{\ }}$= 4600–5400 K, ${T}_{{solid}}$ = 3900 K, ${t}_{{start}}$ = 3.9 Ga, ${T}_{now}$ = 3700 K, and $Ag{e}_{MO}$ = 4.0 Gyr. (c) Pearson and Spearman correlation coefficient matrix between various parameters and CMB cooling rates during mantle overturn; 100 000 simulations are needed to obtain a stable correlation coefficient.

We model the CMB cooling history (Fig. 1b) considering the rapid demise of the basal MO (Fig. 1a) due to mantle overturn. In our framework, the CMB temperature initially decreases approximately linearly over time [20]. According to the evolution of the Archean continents, the rapid demise of the basal MO during mantle overturn can be described by a gamma distribution. Variations in basal MO thickness can be translated into changes in CMB temperature because of the nearly linear relationship between the solidus temperature and depth at the narrow depth range of the lowermost mantle [36] (see details in Methods). Subsequently, the CMB cooled down to its present temperature. The main parameters in the model are: the initial temperature at the CMB ${T}_{{initial}}$, solidus temperature of the MO at the CMB ${T}_{{solid}}$, current temperature at the CMB ${T}_{now}$, lifetime of the basal MO in traditional evolutionary models $Ag{e}_{MO}$ (see Eq. 1), and onset time of mantle overturn ${t}_{{start}}$ (Table 1).

**Table 1.** Parameters used in simulations.

Parameter	Symbol	Values/ranges	Number of increments
Initial temperature at CMB^a^	${T}_{{initial}}$	4150–6200 K	22
Solidus temperature of MO at CMB^b^	${T}_{{solid}}$	3700–4100 K	17
Current temperature at CMB^c^	${T}_{now}$	3500–3700 K	9
Lifetime of the basal MO in traditional evolutionary models^d^	$Ag{e}_{MO}$	2.2–4.5 Gyr	13
Onset time of mantle overturn (upwelling)^e^	${t}_{{start}}$	3.6–3.9 Ga	16
End time of mantle overturn^e^	${t}_{end}$	2.5 Ga	
Core mass	${M}_C$	2.0$\times $10^24^ kg	
Core radius	${R}_C$	3480 km	
Heat capacity correction factor^f^	${X}_C$	1.11	
Specific heat of core^f^	${C}_P$	800 J/(kg°C)	
^40^K half-life^g^	${\tau }_{{{40}}_K}$	1.25$\times $10^9^ year	
^40^K heat production^g^	${H}_{{{40}}_K}$	2.9$\times $10^−5^ W/kg	
^40^K mass concentration^h^	${C}_{{{40}}_K}$	10–100 ppm	
Present adiabatic heat flow^i^	$Q_a^{now}$	3–28 TW	
Adiabatic decay parameter^j^	$\Phi $	0.256	
Adiabatic decay integral^j^	$\bar{e}( \Phi )$	0.8595	
Ohmic dissipation fraction of convective power^j^	${f}_{ohm}$	1	
Scaling prefactor^j^	${c}_1$	1.65	

a [84]

b [36]

c [85]

d [21]

e [32]

f [82]

g [86]

h [47–49]

i [53–55]

j [52]

**Figure 2.**
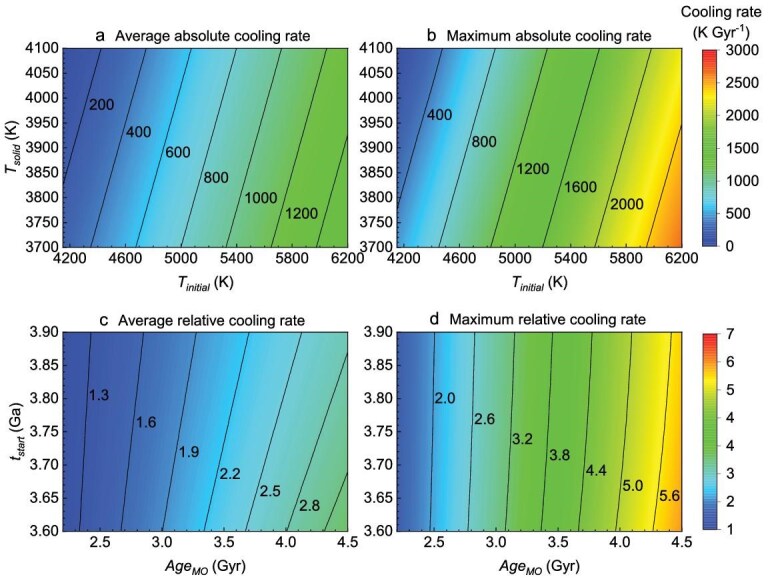
CMB cooling rate during mantle overturn with different parameters. (a) Average absolute cooling rate; (b) maximum absolute cooling rate; (c) average relative cooling rate; (d) maximum relative cooling rate.

To analyze the influence of these uncertain parameters described above on the CMB cooling rate, we generated ∼700 000 thermal histories for a wide range of parameter values derived from previous studies (Table 1). A correlation analysis was then performed to quantify the sensitivity of the CMB cooling rates to these parameters (Fig. 1c). The Pearson and Spearman correlation coefficients between the average cooling rate during mantle overturn and ${T}_{{initial}}$ exceeded 0.90, indicating a strong positive correlation (Fig. 1c). This suggests that ${T}_{{initial}}$ is the most important factor influencing the cooling rate. A higher ${T}_{{initial}}$ corresponds to a higher temperature at the onset of mantle overturn, leading to faster CMB cooling during this event. A longer $Ag{e}_{MO}$ slightly increases the temperature at the onset of mantle overturn, resulting in a weak positive correlation with the average cooling rate. On the other hand, ${t}_{{start}}$ and ${T}_{{solid}}$ exhibit weak negative correlations with the average cooling rate, while ${T}_{now}$ has a negligible effect. The correlation coefficient of the maximum cooling rate is similar to that of the average value. These results suggest that mantle overturn leads to rapid reductions in CMB temperature (Fig. 2a and b) [37,38].

The CMB cooling rate during the mantle overturn, relative to the cooling rate before the overturn, more clearly illustrates the rapid temperature decrease caused by mantle overturn. The average relative cooling rate can be simplified as$\ ( {Ag{e}_{MO} - {t}_{{earth}} + {t}_{{start}}} )/( {{t}_{{start}} - {t}_{end}} )$. Among these parameters, $Ag{e}_{MO}$ is the most influential, with Pearson and Spearman correlation coefficients >0.95 (Fig. 1c), indicating its strong positive correlation with the average relative cooling rate. In contrast, ${t}_{{start}}$ shows a weaker negative correlation with this rate, and other parameters have a negligible influence. The solidification of the basal MO spanned billions of years [20,21], primarily due to the insulating properties of the overlying solid mantle, and the role of the adjacent core as a heat sink. The wide variability in $Ag{e}_{MO}$ is attributable to the poor constraining of the primary influencing factors, such as the melting point and adiabatic gradient [21]. For $Ag{e}_{MO} > $ 2.5 Gyr, mantle overturns will increase the CMB average cooling rate to at least a factor of 1.35 (Fig. 2c), with the maximum relative cooling rate reaching at least 2.0 (Fig. 2d).

### Water-induced mantle overturn increases the CMB heat flow (***Q_C_***)

**Figure 3.**
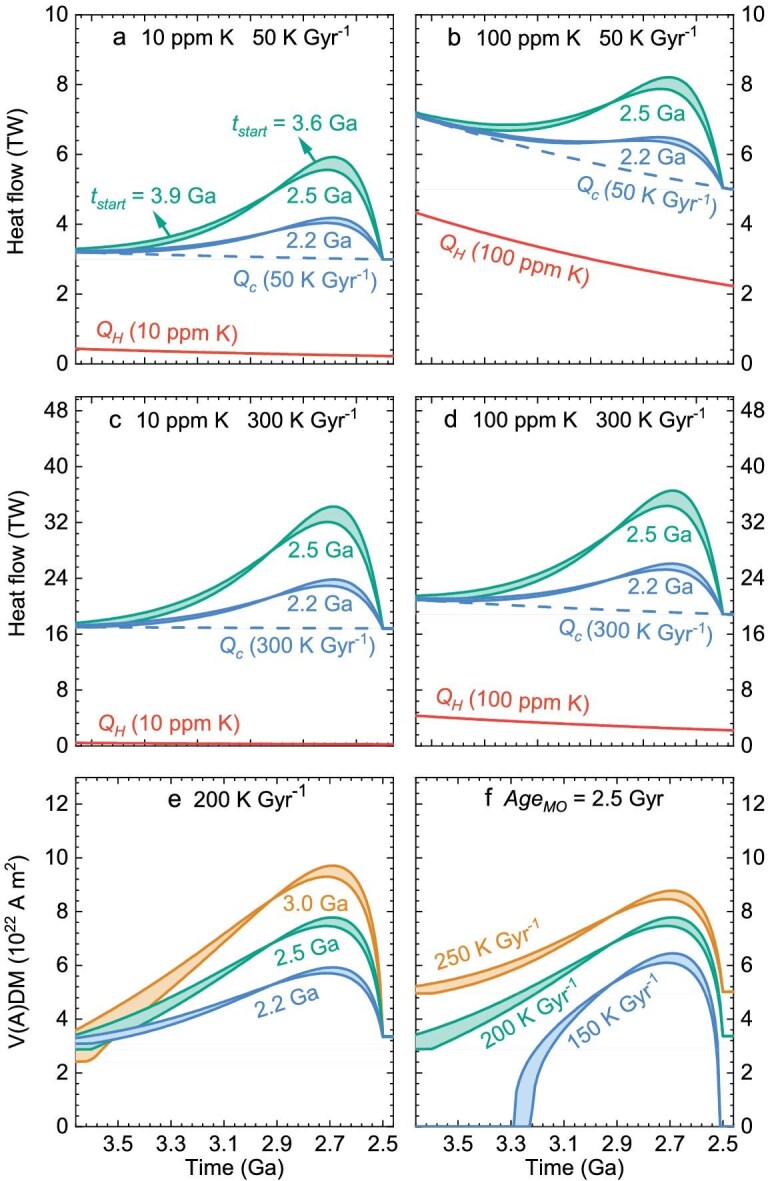
Evolution of CMB heat flow and virtual dipole moment (VDM). (a and c) Heat flows at low radioactive energy contributions. (b and d) Heat flows at high radioactive energy contributions. For panels (a and b): core cooling rate without overturn of 50 K/Gyr, and $Ag{e}_{MO}$ of 2.2–2.5 Gyr. For panels (c and d): core cooling rate of 300 K/Gyr, and $Ag{e}_{MO}$ of 2.2–2.5 Gyr. The red solid curves indicate radioactive energy. (e) VDMs for $Q_a^{now}$ = 9 TW, 10 ppm K, core cooling rate of 200 K/Gyr, and $Ag{e}_{MO}$ = 2.2–3.0 Gyr. (f) VDMs for $Q_a^{now}$ = 9 TW, 10 ppm K, $Ag{e}_{MO}$ = 2.5 Gyr, and core cooling rate of 150–250 K/Gyr.

The MO viscosity is expected to be low due to its high temperature, especially when it contains a certain amount of water, which makes it strongly convective [39]. Meanwhile, the convection turnover time of the Earth’s core is only ∼200 years for a typical convection rate of $5 \times {10}^{ - 4}$ m s^−1^ [40]. Hence, the cooling rates of the Earth’s core, the basal MO, and CMB should be equal [20,41]. Therefore, mantle overturns substantially increase the cooling rate of the Earth’s core (Fig. 2), which increases CMB heat flow ${Q}_C$ owing to energy conservation (see details in Methods). Additionally, the increase in thermal conductivity at the CMB following overturn may also accelerate the cooling of the Earth’s core [42,43]. The parameters used to simulate the ${Q}_C$ evolution are listed in Table 1, where the mass concentration of radioactive elements depends on their partition coefficients between the core and molten mantle. Early studies at low pressures and temperatures found that a few hundred parts per million of potassium (K) may partition into the Earth’s core [44–46]. Under conditions of core formation, experimental and theoretical studies suggest that radioactive elements are highly lithophile, with a K concentration of <50 ppm in the core [47–49]. We show two cases reflecting different radioactive energy contributions: one with 10 ppm K as the minimum radioactive energy contribution, and another with 100 ppm K as the maximum radioactive energy contribution (Fig. 3a–d).

Our results reveal a large increase in ${Q}_C$ during mantle overturn. Specifically, for scenarios with 10 ppm K, the mantle overturn increases ${Q}_C$ by 40% for $Ag{e}_{MO}$ of 2.2 Gyr and core cooling rate (without overturn) of 50 K/Gyr (Fig. 3a). This increase becomes even more pronounced with larger $Ag{e}_{MO}$ and higher core cooling rate, while ${t}_{{start}}$ has a small effect (Fig. 3a–d). Although greater radioactive heat production attenuates this increase, the mantle overturn can still increase ${Q}_C$ by over 20% with 100 ppm K (Fig. 3b).

### The mantle overturn explains the enhanced geodynamo at ∼3.5–2.5 Ga

**Figure 4.**
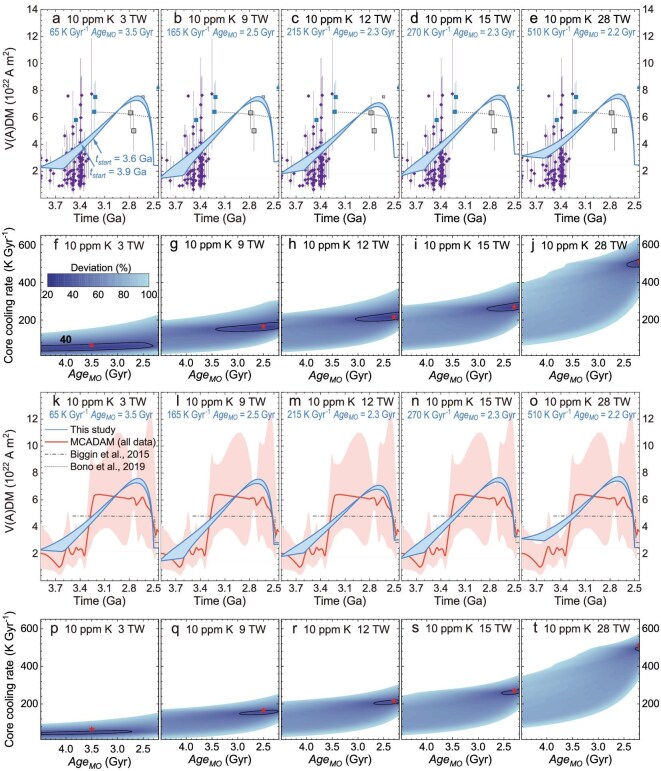
Evolution of virtual dipole moment (VDM) (a-e, k-o) and parameter space of mantle overturns (f-j, p-t). The symbols are compiled from Tarduno *et al.* [13]. The blue and gray squares represent selected Thellier-type (thermal) single-crystal paleointensity and bulk rock studies, respectively. Large squares are time-averaged paleomagnetic dipole moments, whereas small squares indicate VDMs [5,9,57,83]. The purple diamonds represent zircon paleointensity results [3,6,11,12]. Bono *et al.* [5] focused on paleointensity from slow-cooling intrusives (gray dotted curve). The red curves show the results from the Monte Carlo Axial Dipole Average Model (MCADAM) [17] based on all data in the PINT database [16]. The shaded area indicates the 95% confidence interval. Biggin *et al.* [14] obtained smaller variations in VDM around 2.5 Ga based on the earlier PINT database (gray dotted-dash curve). The second and fourth rows show the parameter range of mantle overturn to explain paleointensity records before the end of the Archean. The *x* axis represents $Ag{e}_{MO}$, and the *y* axis represents the core cooling rate without mantle overturn. The colors indicate the time-averaged deviation between the model and records compiled by Tarduno *et al.* [13] (second row) or results of MCADAM (fourth row). The deviation from the results by Tarduno *et al.* [13] before ∼3.3 Ga is calculated as the mean difference between the model and zircon paleointensity records within 0.05 Ga [3,6,11,12], whereas the deviation after ∼3.3 Ga is calculated as the difference between the model and Bono *et al.* [5]. The red stars indicate the parameter settings used in the models for first and third rows.

The geodynamo is highly sensitive to CMB conditions, especially regarding thermal state and heat flow [8,30,50,51]. According to the scaling laws proposed by Aubert *et al.* [52], the value of the virtual dipole moment (VDM) is proportional to ${( {{Q}_C - {Q}_a} )}^{0.34}$. Hence, high ${Q}_C$ during mantle overturn generates a stronger dynamo, potentially explaining paleointensity records around 3.5–2.5 Ga [3,6,11–13,17]. Moreover, accelerated core cooling during mantle overturn promotes the exsolution of light elements, which may further increase the magnetic field strength, but this study does not consider this due to the uncertain exsolution time and rate [26]. We estimate the effect of mantle overturn on VDM, using the parameters listed in Table 1 (see details in Methods). Adiabatic heat flow ${Q}_a$ depends on the thermal conductivity of the Earth’s core, which diverges drastically, and ranges from 20 to 226 Wm^−1^K^−1^ [53,54]. The correction for sample thickness reduces the maximum value of 226 to 185 Wm^−1^K^−1^ [55], resulting in a present adiabatic heat flow $Q_a^{now}$ range of 3−28 TW. We consider the effect of temperature on CMB heat flow ${Q}_a$ because temperature increases the conductivity of iron alloys [56]. Our results demonstrate that higher core cooling rates (without overturn) lead to an overall increase in ${Q}_C$ and VDM, while larger $Ag{e}_{MO}$ effectively increase them during mantle overturn (Fig. 3). Therefore, for a wide range of $Q_a^{now}$, mantle overturn can well explain the observed increase in magnetic field strength during the Archean by adjusting the core cooling rate and $Ag{e}_{MO}$ (Fig. 4 for 10 ppm K, and Fig. S1 for 100 ppm K). A larger $Q_a^{now}$ requires a higher core cooling rate and generally favors a smaller $Ag{e}_{MO}.$ For $Ag{e}_{MO}\ $of ∼2.3 Gyr, the model can match the Archean paleointensity no matter what the $Q_a^{now}$ (Fig. 4 and Fig. S1). Better constraints on the Archean paleointensity and adiabatic heat flow $Q_a^{now}\ $are therefore crucial for evaluating $Ag{e}_{MO}$ and the core cooling rate. The paleointensity records and model predictions are independent, and their agreement provides a validation of both. It is worth noting, the paleointensity records compiled by Tarduno *et al.* [13] indicate that this strong geomagnetic field persisted until ∼2.0 Ga according to a time-averaged single-crystal paleointensity [57]. If this reflects a more realistic paleomagnetic signal, additional mechanisms would be required to sustain this strong geodynamo after 2.5 Ga. One possibility is the onset of plate tectonics, cold subducting slabs reaching the CMB could accelerate its cooling and modify the heat flux pattern, both of which could influence the geodynamo [13,30].

The onset of plate tectonics may indeed have occurred at the end of the Archean [34,58–61]. The upwelling mantle overturn is balanced by downwelling and may induce local subduction, as suggested by geodynamic simulations [62]. Other factors, such as the density difference between the continental and oceanic crusts, very dense banded iron formation at the continental margin, spreading of buoyant continents, and lubricating continental sediment, all favor subduction [63–66]. Therefore, with the growth of continental crust by mantle overturn, subduction also develops. The evidence for subduction has been found at the Paleoarchean or earlier [67–70], but they are likely local subduction. The Ni/Co and Cr/Zn ratios that track the bulk MgO composition of the Archean upper continental crust decrease with time from ∼3.2 to 2.5 Ga [71]. During the same period, the Rb/Sr ratio that tracks the thickness of the continental crust increases [72]. The changes in these ratios may reflect that the subduction has been significant around 3.0 Ga, but plate tectonics probably initiated later because of the demanding global feature of plate tectonics. The formation mechanism of continental crust by mantle overturns are no longer applicable once mantle overturns exhaust the basal MO. Therefore, the formation mechanism of continental crust must change from the dominance of mantle-overturn to subduction over time. The change of the dominant formation mechanism of the continental crust should be able to generate a global change in the rock properties of that crust. Global change has indeed been extensively observed at the end of the Archean [73,74]. Moreover, as the hallmark of plate tectonics, temporally and spatially associated rock assemblages with mid-ocean ridge and supra-subduction-zone origins are indeed found around 2.5 Ga [75]. Therefore, the onset of plate tectonics, likely occurring at the end of the Archean, may help explain the high paleointensity that persisted until around 2.0 Ga [13,57].

**Figure 5.**
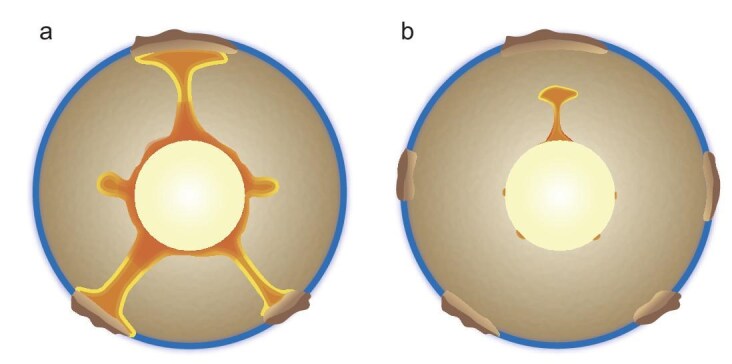
Schematic of mantle overturn. (a) During mantle overturn, large-scale plumes rise toward the surface, contributing to the formation of the Archean continents, while the CMB temperature rapidly decreases as magma is removed, substantially enhancing the geodynamo at that time. (b) At the end of mantle overturn, the CMB and core return to a relatively stable state.

Water-induced mantle overturns [32] lasted for billions of years and traversed the whole mantle. The temporal and spatial scales of mantle overturns are comparable to those of plate tectonics. But we know very little about many aspects of the mantle overturns. For example, how did the mantle overturn evolve with time? As shown in Wu *et al.* [32] and this study, water-induced mantle overturns had various effects on the early Earth, such as the formation of TTG and the paleomagnetic field in the Archean. These effects are very useful in constraining the processes of mantle overturns. We estimate thickness variations of the basal MO in Fig. 1a by taking account of the growing emergence of TTG after around 3.6 Ga and the disappearance of TTG at the end of the Archean (see details in Methods). The resulting VDM in Fig. 4 is quite similar to the thickness variation in Fig. 1a, suggesting that VDM has the potential to provide fundamental constraints on the mantle overturns. The consistency between this study and paleointensity records in Fig. 4 indicates that two effects of the mantle overturn, namely, the formation of TTG and paleomagnetic field, generate a concordant picture on the evolution of mantle overturn (Fig. 5). We believe that our findings would stimulate three-dimensional geodynamic simulations on mantle overturn and its effect on the geodynamo.

## METHODS

Wu *et al.* [32] showed that the basal MO contained only limited water throughout most of its crystallization, but experienced a sharp increase in water content during the late stage. Due to the inefficiency of water diffusion into the core and water becoming less siderophile at high concentrations [76,77], a substantial amount of water accumulated in the basal MO at the end of crystallization, rendering it gravitationally unstable and triggering mantle overturns. Within reasonable parameter ranges, mantle overturn could occur during the Archean [32]. To investigate the effect of mantle overturn on the geomagnetic field, we modeled the CMB cooling history, and then derived the CMB heat flow history through energy conservation. Finally, the VDM was calculated using the scaling laws in [52].

### CMB cooling history

The temperature of basal MO decreases approximately linearly with time before water-induced mantle overturn (Fig. 1b) [20]; the CMB cooling rate before mantle overturn can be expressed as


(1) \begin{eqnarray*}
\frac{{dT\!\left( t \right)}}{{dt}} = \frac{{{T}_{{initial}} - {T}_{{solid}}}}{{Ag{e}_{MO}}},
\end{eqnarray*}

where *t* is time relative to the present, ${T}_{{initial}}$ is the initial temperature at the CMB, ${T}_{{solid}}$ is the solidus temperature of the MO at the CMB, and $Ag{e}_{MO}$ is the lifetime of the basal MO in traditional evolutionary models without mantle overturn.

The thickness of the basal MO and hence the CMB temperature is also affected by the upwelling of basal MO [32]. In the late stages of crystallization, the basal MO becomes less dense than the overlying mantle because of water enrichment, which triggers mantle overturn. Before the basal MO becomes gravitationally unstable, its upwelling is possible because density heterogeneity may cause a small local area in the basal MO to be less dense than the overlying mantle [32]. The probability of upwelling depends on the density difference between the basal MO and overlying mantle. The density difference first increases with crystallization because of the incompatibility of iron and limited water content in the basal MO, and then decreases with crystallization because of water enrichment [32]. The maximum density difference occurs around 3.6 Ga if the time that the basal MO becomes gravitationally unstable is fixed at 2.9 Ga [32]. The increase in density difference hinders the formation of upwelling up to around 3.6 Ga. The limited water content in these upwellings explains why TTG is rare in the Hadean. After the density difference reaches the maximum at ∼3.6 Ga, more and more upwellings can form with time and these upwellings contain a significant amount of water, which is consistent with the rarity of TTG and the lithospheric mantle with age >3.6 Ga, as well as with the growing emergence of TTG and lithospheric mantle with age <3.6 Ga (Fig. 1b) [34,78,79]. Therefore, the thickness of the basal MO decreases slowly around 3.6 Ga followed by an accelerated decrease around 2.9 Ga. Mantle overturns probably run out of the basal MO at the end of the Archean. Thus, we employ the gamma distribution function with shape parameter $\alpha = 2$ and scale parameter $\beta = 0.2$ to describe the decrease in thickness due to the mantle overturn (Fig. 1a), which could also be used to describe the CMB temperature decrease due to the mantle overturn, given the nearly linear relation between the solidus temperature and depth at the narrow depth range of the lowermost mantle [36]. The cooling rate during mantle overturn can therefore be expressed as


(2) \begin{eqnarray*}
\frac{{dT\!\left( t \right)}}{{\ dt}} &=& \frac{{\left( {{T}_{{start}} - {T}_{{solid}}} \right)}}{{{H}_{{start}}}}\frac{{dH}}{{dt}} + \frac{{dT\!\left( {{t}_{{start}}} \right)}}{{\ dt}}\nonumber\\
&=& a\frac{1}{{{\mathrm{\Gamma }}\!\left( \alpha \right){\beta }^\alpha }}{\left( {t - b} \right)}^{\alpha - 1}{e}^{ - \frac{{t - b}}{\beta }} + c,\nonumber\\
\end{eqnarray*}

where $\frac{{dH}}{{dt}}$ is the decrease rate in the thickness of basal MO due to the mantle overturn, ${H}_{{start}}$ and ${T}_{{start}}$ are the thicknesses of the basal MO and CMB temperature at the onset of mantle overturn, respectively; *a, b* and *c* can be obtained as follows by ensuring that the CMB cooling rate and temperature are continuous:


(3) \begin{eqnarray*}
\left\{ {\begin{array}{@{}*{1}{c}@{}} {\frac{{dT\left( {{t}_{end}} \right)}}{{dt}} = \frac{{{T}_{{solid}} - {T}_{now}}}{{{t}_{end}}}},\\ {\frac{{dT\left( {{t}_{{start}}} \right)}}{{dt}} = \frac{{{T}_{{initial}} - {T}_{{solid}}}}{{Ag{e}_{MO}}}},\\ {\mathop \int \nolimits_{{t}_{end}}^{{t}_{{start}}} \left( {\frac{{dT\left( t \right)}}{{dt}}} \right)dt = \frac{{\left( {{T}_{{initial}} - {T}_{{solid}}} \right)\left( {{t}_{{start}} + Ag{e}_{MO} - {t}_{{earth}}} \right)}}{{Ag{e}_{MO}}}}, \end{array}} \right.
\end{eqnarray*}

where ${T}_{now}$ is the current temperature at the CMB, ${t}_{{start}}$ is the onset time of mantle overturn, ${t}_{end}\ $is the end time of mantle overturn, which inferred to be ∼2.5 Ga [32], and ${t}_{{earth}}{\mathrm{\ }}$is Earth’s age. Finally, the CMB temperature gradually decreases to its present value with the cooling rate given by


(4) \begin{eqnarray*}
\frac{{dT\!\left( t \right)}}{{dt}} = \frac{{{T}_{{solid}} - {T}_{now}}}{{{t}_{end}}}.
\end{eqnarray*}

### CMB heat flow

The gravitational energy resulting from core density changes over time is retained as compressional energy, and thus does not contribute to heat balance [80]. Therefore, the CMB heat flow before crystallization of the inner core can be expressed as [81]


(5) \begin{eqnarray*}
{Q}_C = {X}_C{C}_P{M}_C\frac{{d{T}_C}}{{dt}} + {Q}_H,
\end{eqnarray*}

where ${X}_C$ is the heat capacity correction factor [82], ${C}_P$ is the specific heat of the core,$\ {T}_C$ is the temperature of the Earth’s core, and ${Q}_H$ is the radioactive energy, given by


(6) \begin{eqnarray*}
{Q}_H = {M}_{\mathrm{C}} {\sum}_e {C}_e {H}_e {\mathrm{exp}} \left( {\frac{{t{\mathrm{log\, }} 2}}{{{\tau }_e}}} \right),
\end{eqnarray*}

with ${C}_e$ being the mass concentration of radioactive element *e*, ${H}_e$ and ${\tau }_e$ being the associated heat release and half-life, respectively.

### VDM

The VDM before crystallization of the inner core can be estimated using the scaling laws proposed by Aubert *et al.* [52] as follows:


(7) \begin{eqnarray*}
M = \frac{{4\pi {R}^3}}{{\sqrt 2 \mu }}\frac{{{B}_{{\mathrm{rms}}}}}{{{b}_{{\mathrm{dip}}}}},
\end{eqnarray*}

where *μ* is the magnetic permeability. The rms core magnetic field ${B}_{{\mathrm{rms}}}$ can be expressed as


(8) \begin{eqnarray*}
{B}_{{\mathrm{rms}}} = {c}_1f_{ohm\ }^{0.5}{p}^{0.34}{(\rho \mu )}^{1/2}{\mathrm{\Omega }}{R}_C,
\end{eqnarray*}

where ${c}_1$ is a scaling pre-factor, ${f}_{ohm}$ is the ohmic dissipation fraction of convective power, $\rho $ is the core density, and ${\mathrm{\Omega }}$ is the Earth’s rotation rate. Convective power *p* can be expressed as


(9) \begin{eqnarray*}
p = \left( {{Q}_C - {Q}_a} \right){\epsilon }_S,
\end{eqnarray*}

(10) \begin{eqnarray*}
{\epsilon }_S = {e}^\Phi \bar{e}\left( \Phi \right) - 1,
\end{eqnarray*}

where ${Q}_a$ is the adiabatic heat flow, ${\epsilon }_S$ is the thermodynamic efficiency of thermal convection, ${\mathrm{\Phi }}$ and $\bar{e}( {\mathrm{\Phi }} )$ are the adiabatic decay parameter and adiabatic decay integral, respectively.

## Supplementary Material

nwaf578_Supplemental_File

## Data Availability

All the data used are available within the article.
