# Supplementary material for: Water-induced mantle overturn explains high Archean paleointensities
Source: Natl Sci Rev. 2025 Dec 23;13(3):nwaf578. doi: 10.1093/nsr/nwaf578 (PMC12887327; doi:10.1093/nsr/nwaf578)
Supplement: nwaf578_Supplemental_File [file nwaf578_supplemental_file.docx]

Supplementary Materials for

**Water-Induced Mantle Overturn Explains High Archean Paleointensities**

Dong Wang ^a^, Zhongqing Wu ^a,b*^

^a^ State Key Laboratory of Precision Geodesy, School of Earth and Space Sciences, University of Science and Technology of China, Hefei 230026, China

^b^ Mengcheng National Geophysical Observatory, University of Science and Technology of China, Mengcheng, 233500, China

^*^ Corresponding author. Email: [wuzq10@ustc.edu.cn](mailto:wuzq10@ustc.edu.cn)

**This file includes:**

Fig. S1-S2





**Fig. S1 |** **Evolution of virtual dipole moment (VDM) (first and third rows)** **and parameter space of mantle overturns (second and fourth rows)**. The symbols are compiled from Tarduno et al. [1]. The blue and gray squares represent selected Thellier-type (thermal) single-crystal paleointensity and bulk rock studies, respectively. Large squares are time-averaged paleomagnetic dipole moments, whereas small squares indicate VDMs [2-5]. The purple diamonds represent zircon paleointensity results [6-9]. Bono et al. [2] focused on paleointensity from slow-cooling intrusives (gray dotted curve). The red curves show the results from the Monte Carlo Axial Dipole Average Model (MCADAM) [10] based on all data in the PINT database [11]. The shaded area indicates the 95% confidence interval. Biggin et al. [12] obtained smaller variations in VDM around 2.5 Ga based on the earlier PINT database (gray dotted-dash curve). The second and fourth rows show the parameter range of mantle overturn to explain paleointensity records before the end of the Archean. The *x* axis represents ${Age}_{MO}$, and the *y* axis represents the core cooling rate without mantle overturn. The colors indicate the time-averaged deviation between the model and records compiled by Tarduno et al. [1] (second row) or results of MCADAM (fourth row). The deviation from the results by Tarduno et al. [1] before ~3.3 Ga is calculated as the mean difference between the model and zircon paleointensity records within 0.05 Ga [6-9], whereas the deviation after ~3.3 Ga is calculated as the difference between the model and Bono et al. [2]. The red stars indicate the parameter settings used in the models for first and third rows.





**Fig. S2 | Evolution of virtual dipole moment (VDM).** The red curves are from the Monte Carlo Axial Dipole Average Model (MCADAM) [10] based on the PINT database [11], using reliable data with paleointensity quality $Q_{PI}\geq3$, or reliable data with strictest criteria ($Q_{AGE}$ + $Q_{ALT}$ + $Q_{MD}$). Shaded area denotes the 95% confidence interval. Biggin et al. [12] yielded smaller variations in VDM values at ~2.5 Ga based on the earlier PINT database (gray dotted-dash curve).

**Reference**

1. Tarduno JA, Zhou T, Huang W*, et al*. Earth’s magnetic field and its relationship to the origin of life, evolution and planetary habitability. *Natl Sci Rev* 2025; **12**:

2. Bono RK, Tarduno JA, Nimmo F*, et al*. Young inner core inferred from Ediacaran ultra-low geomagnetic field intensity. *Nat Geosci* 2019; **12**:143-147.

3. Zhou T, Tarduno JA, Nimmo F*, et al*. Early Cambrian renewal of the geodynamo and the origin of inner core structure. *Nat Commun* 2022; **13**:4161.

4. Huang W, Tarduno JA, Zhou T*, et al*. Near-collapse of the geomagnetic field may have contributed to atmospheric oxygenation and animal radiation in the Ediacaran Period. *Commun Earth Environ* 2024; **5**:207.

5. Zhou T, Ibañez-Mejia M, Bono RK*, et al*. Magnetization and age of ca. 544 Ma syenite, eastern Canada: Evidence for renewal of the geodynamo. *Earth Planet Sci Lett* 2024; **639**:118758.

6. Tarduno JA, Cottrell RD, Bono RK*, et al*. Paleomagnetism indicates that primary magnetite in zircon records a strong Hadean geodynamo. *Proc Natl Acad Sci USA* 2020; **117**:2309-2318.

7. Tarduno JA, Cottrell RD, Davis WJ*, et al*. A Hadean to Paleoarchean geodynamo recorded by single zircon crystals. *Science* 2015; **349**:521-524.

8. Tarduno J, Cottrell R, Bono R*, et al*. Hadean to Eoarchean stagnant lid tectonics recorded by the paleomagnetism of zircons. Abstract-10278, EGU General Assembly, Vienna, 23-28 April 2023.

9. Tarduno JA, Cottrell RD, Bono RK*, et al*. Hadaean to Palaeoarchaean stagnant-lid tectonics revealed by zircon magnetism. *Nature* 2023; **618**:531-536.

10. Bono RK, Paterson GA, Biggin AJ. MCADAM: A Continuous Paleomagnetic Dipole Moment Model for at Least 3.7 Billion Years. *Geophys Res Lett* 2022; **49**:e2022GL100898.

11. Bono RK, Paterson GA, van der Boon A*, et al*. The PINT database: a definitive compilation of absolute palaeomagnetic intensity determinations since 4 billion years ago. *Geophys J Int* 2021; **229**:522-545.

12. Biggin AJ, Piispa EJ, Pesonen LJ*, et al*. Palaeomagnetic field intensity variations suggest Mesoproterozoic inner-core nucleation. *Nature* 2015; **526**:245-248.
